# {4-Bromo-2-[2-(isopropyl­amino)ethyl­imino­meth­yl]phenolato}thio­cyanato­copper(II)

**DOI:** 10.1107/S1600536808010404

**Published:** 2008-04-18

**Authors:** Jun-Ying Ma, Yin-Ting He

**Affiliations:** a Chemical Engineering & Pharmaceutics College, Henan University of Science and Technology, Luoyang Henan 471003, People’s Republic of China, and, Department of Chemistry, Pingdingshan University, Pingdingshan Henan 467000, People’s Republic of China; b Zhoukou Vocational and Technical College, Zhoukou Henan 466600, People’s Republic of China

## Abstract

In the title mononuclear Schiff base copper(II) complex, [Cu(C_12_H_16_BrN_2_O)(NCS)], the Cu^II^ ion is coordinated by two N atoms and one O atom from a Schiff base ligand, and by one N atom from a thio­cyanate anion, giving a square-planar geometry. There are long-range inter­actions between the Cu atom and S [3.151 (5) Å] and Br [3.929 (5) Å] atoms above and below the square plane.

## Related literature

For related literature, see: Ma *et al.* (2005 ▶); Ma, Gu *et al.* (2006 ▶); Ma, Lv *et al.* (2006 ▶); Ma, Wu *et al.* (2006 ▶).
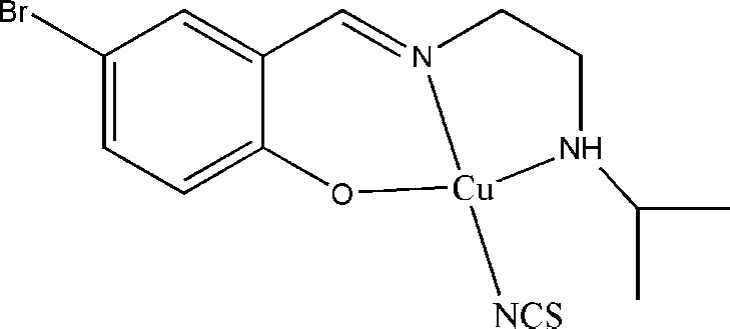


## Experimental

### 

#### Crystal data

[Cu(C_12_H_16_BrN_2_O)(NCS)]
*M* *_r_* = 405.80
Monoclinic, 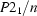

*a* = 6.161 (2) Å
*b* = 20.223 (3) Å
*c* = 12.930 (3) Å
β = 95.332 (5)°
*V* = 1604.0 (7) Å^3^
*Z* = 4
Mo *K*α radiation
μ = 3.98 mm^−1^
*T* = 298 (2) K
0.40 × 0.38 × 0.37 mm

#### Data collection

Bruker SMART 1000 diffractometer
Absorption correction: multi-scan (*SADABS*; Sheldrick, 1996 ▶) *T*_min_ = 0.299, *T*_max_ = 0.321 (expected range = 0.214–0.229)
11914 measured reflections
3474 independent reflections
2126 reflections with *I* > 2σ(*I*)
*R*_int_ = 0.076

#### Refinement

*R*[*F* ^2^ > 2σ(*F* ^2^)] = 0.055
*wR*(*F* ^2^) = 0.153
*S* = 1.01
3474 reflections
183 parameters
H-atom parameters constrained
Δρ_max_ = 0.81 e Å^−3^
Δρ_min_ = −0.49 e Å^−3^

### 

Data collection: *SMART* (Bruker, 2007 ▶); cell refinement: *SAINT* (Bruker, 2007 ▶); data reduction: *SAINT*; program(s) used to solve structure: *SHELXS97* (Sheldrick, 2008 ▶); program(s) used to refine structure: *SHELXL97* (Sheldrick, 2008 ▶); molecular graphics: *SHELXTL* (Sheldrick, 2008 ▶); software used to prepare material for publication: *SHELXL97*.

## Supplementary Material

Crystal structure: contains datablocks global, I. DOI: 10.1107/S1600536808010404/om2226sup1.cif

Structure factors: contains datablocks I. DOI: 10.1107/S1600536808010404/om2226Isup2.hkl

Additional supplementary materials:  crystallographic information; 3D view; checkCIF report

## Figures and Tables

**Table:** 

Cu1—O1	1.903 (4)
Cu1—N1	1.932 (4)
Cu1—N3	1.959 (5)
Cu1—N2	2.075 (5)

**Table:** 

O1—Cu1—N1	92.32 (17)
O1—Cu1—N3	87.98 (19)
N1—Cu1—N3	177.5 (2)
O1—Cu1—N2	171.59 (18)
N1—Cu1—N2	84.45 (18)
N3—Cu1—N2	94.91 (19)

**Fig. 1.**
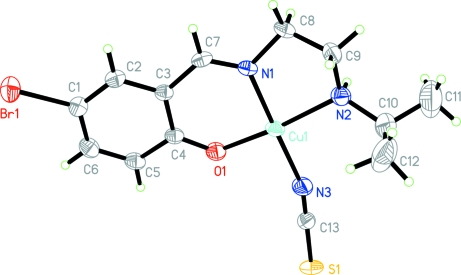
The molecular structure at the 30% probability level ellipsoids.

## supplementary crystallographic information

### Comment

Recently, we have reported some metal complexes derived from the Schiff base ligands (Ma, Lv *et al.*, 2006; Ma, Gu *et al.*, 2006; Ma, Wu *et al.*, 2006; Ma *et al.*, 2005). As part of a further investigation of the structures of such complexes, the title mononuclear copper(II) complex, is reported in this paper.

In the complex the Cu atom is coordinated by two nitrogen atoms and one oxygen atom from a Schiff base ligand, and by one nitrogen atom from a thiocyanate anion, giving a square planar geometry (Fig. 1). There exist long range interactions between the Cu and S (3.151 (5) Å; symmetry code: 1 + x, y, z) and Br (3.929 (5) Å; symmetry code: 1 - x, - y, 1 - z) atoms above and below the square plane. All the bond lengths and angles (Table 1) related to the Cu atom in the complex are within normal ranges. The four coordinating atoms around the Cu centre are approximately coplanar, giving a square-planar geometry with an average deviation of 0.047 (4) Å; the Cu atom lies 0.089 (2) Å above this plane. The C8—C9—N2—C10 torsion angle is 2.0 (3)°.

### Experimental

*N*-Isopropylethane-1,2-diamine (0.5 mmol, 51.0 mg) and 5-bromosalicylaldehyde (0.5 mmol, 100.5 mg) were dissolved in methanol (30 ml). The mixture was stirred for 1 h to obtain a clear yellow solution. To the solution was added with stirring a methanol solution (20 ml) of copper(II) acetate (0.5 mmol, 99.6 mg) and a methanol solution (10 ml) of ammonium thiocyanate (0.5 mmol, 38.0 mg). After keeping the resulting solution in air for a few days, blue block-shaped crystals were formed.

### Refinement

All H atoms were placed in geometrically idealized positions and constrained to ride on their parent atoms with C—H = 0.93-0.97 Å, N—H = 0.91 Å, and with Uĩso~(H) = 1.2U~eq~(C,N) and 1.5U~eq~(methyl C).

### Crystal data

**Table:**

[Cu(C_12_H_16_BrN_2_O)(NCS)]	*F*_000_ = 812
*M**_r_* = 405.80	*D*_x_ = 1.680 Mg m^−^^3^
Monoclinic, *P*2_1_/*n*	Mo *K*α radiation λ = 0.71073 Å
Hall symbol: -P 2yn	Cell parameters from 1880 reflections
*a* = 6.161 (2) Å	θ = 2.5–24.3º
*b* = 20.223 (3) Å	µ = 3.98 mm^−^^1^
*c* = 12.930 (3) Å	*T* = 298 (2) K
β = 95.332 (5)º	Block, blue
*V* = 1604.0 (7) Å^3^	0.40 × 0.38 × 0.37 mm
*Z* = 4	

### Data collection

**Table:**

Bruker SMART 1000 diffractometer	3474 independent reflections
Radiation source: fine-focus sealed tube	2126 reflections with *I* > 2σ(*I*)
Monochromator: graphite	*R*_int_ = 0.076
*T* = 298(2) K	θ_max_ = 27.0º
ω scans	θ_min_ = 1.9º
Absorption correction: multi-scan(SADABS; Sheldrick, 1996)	*h* = −7→7
*T*_min_ = 0.299, *T*_max_ = 0.321	*k* = −25→25
11914 measured reflections	*l* = −16→16

### Refinement

**Table:**

Refinement on *F*^2^	Secondary atom site location: difference Fourier map
Least-squares matrix: full	Hydrogen site location: inferred from neighbouring sites
*R*[*F*^2^ > 2σ(*F*^2^)] = 0.055	H-atom parameters constrained
*wR*(*F*^2^) = 0.153	*w* = 1/[σ^2^(*F*_o_^2^) + (0.0682*P*)^2^] where *P* = (*F*_o_^2^ + 2*F*_c_^2^)/3
*S* = 1.01	(Δ/σ)_max_ < 0.001
3474 reflections	Δρ_max_ = 0.81 e Å^−^^3^
183 parameters	Δρ_min_ = −0.49 e Å^−^^3^
Primary atom site location: structure-invariant direct methods	Extinction correction: none

### Special details

**Table:**

Geometry. All e.s.d.'s (except the e.s.d. in the dihedral angle between two l.s. planes) are estimated using the full covariance matrix. The cell e.s.d.'s are taken into account individually in the estimation of e.s.d.'s in distances, angles and torsion angles; correlations between e.s.d.'s in cell parameters are only used when they are defined by crystal symmetry. An approximate (isotropic) treatment of cell e.s.d.'s is used for estimating e.s.d.'s involving l.s. planes.
Refinement. Refinement of *F*^2^ against ALL reflections. The weighted *R*-factor *wR* and goodness of fit *S* are based on *F*^2^, conventional *R*-factors *R* are based on *F*, with *F* set to zero for negative *F*^2^. The threshold expression of *F*^2^ > σ(*F*^2^) is used only for calculating *R*-factors(gt) *etc*. and is not relevant to the choice of reflections for refinement. *R*-factors based on *F*^2^ are statistically about twice as large as those based on *F*, and *R*- factors based on ALL data will be even larger.

### Fractional atomic coordinates and isotropic or equivalent isotropic displacement parameters (Å^2^)

**Table:**

	*x*	*y*	*z*	*U*_iso_*/*U*_eq_	
Cu1	0.26493 (10)	0.20760 (3)	0.47898 (5)	0.0450 (2)	
N1	0.5364 (7)	0.1721 (2)	0.5425 (3)	0.0408 (10)	
N2	0.3292 (7)	0.2854 (2)	0.5819 (4)	0.0534 (12)	
H2A	0.2664	0.2729	0.6397	0.064*	
N3	−0.0164 (8)	0.2425 (3)	0.4201 (4)	0.0616 (14)	
O1	0.2003 (6)	0.1286 (2)	0.4022 (3)	0.0551 (10)	
S1	−0.4273 (2)	0.25260 (9)	0.31058 (11)	0.0566 (4)	
Br1	0.80195 (10)	−0.08910 (3)	0.28530 (5)	0.0677 (3)	
C1	0.6219 (9)	−0.0194 (3)	0.3274 (4)	0.0475 (13)	
C2	0.6939 (9)	0.0269 (3)	0.3994 (4)	0.0466 (13)	
H2	0.8364	0.0245	0.4299	0.056*	
C3	0.5578 (8)	0.0781 (2)	0.4285 (4)	0.0382 (12)	
C4	0.3387 (8)	0.0829 (3)	0.3822 (4)	0.0418 (12)	
C5	0.2699 (9)	0.0321 (3)	0.3099 (4)	0.0532 (15)	
H5	0.1270	0.0326	0.2795	0.064*	
C6	0.4065 (10)	−0.0175 (3)	0.2836 (5)	0.0552 (15)	
H6	0.3554	−0.0499	0.2365	0.066*	
C7	0.6414 (9)	0.1221 (3)	0.5099 (4)	0.0436 (12)	
H7	0.7808	0.1140	0.5413	0.052*	
C8	0.6326 (9)	0.2117 (3)	0.6316 (4)	0.0544 (15)	
H8A	0.7902	0.2077	0.6376	0.065*	
H8B	0.5801	0.1957	0.6955	0.065*	
C9	0.5675 (9)	0.2829 (3)	0.6140 (4)	0.0536 (15)	
H9A	0.6006	0.3081	0.6773	0.064*	
H9B	0.6478	0.3019	0.5602	0.064*	
C10	0.2371 (15)	0.3525 (4)	0.5575 (6)	0.092 (2)	
H10	0.0818	0.3447	0.5376	0.111*	
C11	0.242 (2)	0.3952 (5)	0.6475 (9)	0.150 (4)	
H11A	0.2122	0.3697	0.7072	0.225*	
H11B	0.1328	0.4290	0.6354	0.225*	
H11C	0.3828	0.4154	0.6596	0.225*	
C12	0.319 (2)	0.3811 (5)	0.4651 (7)	0.152 (5)	
H12A	0.2246	0.4165	0.4396	0.228*	
H12B	0.3221	0.3477	0.4125	0.228*	
H12C	0.4633	0.3979	0.4824	0.228*	
C13	−0.1851 (9)	0.2472 (3)	0.3741 (4)	0.0457 (13)	

### Atomic displacement parameters (Å^2^)

**Table:**

	*U*^11^	*U*^22^	*U*^33^	*U*^12^	*U*^13^	*U*^23^
Cu1	0.0348 (4)	0.0592 (5)	0.0396 (4)	0.0019 (3)	−0.0042 (3)	−0.0043 (3)
N1	0.038 (2)	0.048 (3)	0.035 (2)	−0.006 (2)	−0.0057 (18)	0.002 (2)
N2	0.051 (3)	0.066 (3)	0.044 (3)	0.010 (2)	0.007 (2)	−0.005 (2)
N3	0.042 (3)	0.079 (4)	0.061 (3)	0.011 (3)	−0.008 (2)	−0.015 (3)
O1	0.036 (2)	0.066 (3)	0.061 (3)	0.0002 (19)	−0.0087 (17)	−0.011 (2)
S1	0.0407 (8)	0.0800 (11)	0.0466 (8)	−0.0024 (8)	−0.0086 (6)	0.0161 (8)
Br1	0.0639 (5)	0.0571 (4)	0.0838 (5)	−0.0015 (3)	0.0157 (4)	−0.0128 (3)
C1	0.043 (3)	0.045 (3)	0.055 (3)	−0.004 (3)	0.007 (3)	0.000 (3)
C2	0.043 (3)	0.046 (3)	0.050 (3)	−0.002 (3)	−0.001 (2)	0.007 (3)
C3	0.039 (3)	0.038 (3)	0.037 (3)	−0.004 (2)	0.000 (2)	0.005 (2)
C4	0.037 (3)	0.046 (3)	0.041 (3)	−0.006 (2)	−0.001 (2)	0.004 (2)
C5	0.040 (3)	0.062 (4)	0.054 (4)	−0.009 (3)	−0.011 (3)	−0.007 (3)
C6	0.058 (4)	0.052 (4)	0.054 (4)	−0.013 (3)	0.001 (3)	−0.007 (3)
C7	0.038 (3)	0.048 (3)	0.044 (3)	0.001 (3)	−0.003 (2)	0.015 (3)
C8	0.049 (3)	0.063 (4)	0.048 (3)	−0.006 (3)	−0.013 (3)	−0.009 (3)
C9	0.056 (4)	0.057 (4)	0.049 (3)	−0.002 (3)	0.006 (3)	−0.014 (3)
C10	0.121 (7)	0.082 (5)	0.073 (5)	0.032 (5)	0.002 (5)	−0.017 (4)
C11	0.248 (14)	0.083 (7)	0.124 (9)	0.029 (7)	0.039 (9)	−0.018 (6)
C12	0.280 (15)	0.099 (7)	0.084 (6)	0.089 (9)	0.055 (8)	0.028 (6)
C13	0.046 (3)	0.049 (3)	0.043 (3)	0.002 (3)	0.007 (3)	0.002 (3)

### Geometric parameters (Å, °)

**Table:**

Cu1—O1	1.903 (4)	C4—C5	1.426 (7)
Cu1—N1	1.932 (4)	C5—C6	1.372 (8)
Cu1—N3	1.959 (5)	C5—H5	0.9300
Cu1—N2	2.075 (5)	C6—H6	0.9300
N1—C7	1.292 (6)	C7—H7	0.9300
N1—C8	1.481 (6)	C8—C9	1.507 (8)
N2—C9	1.489 (7)	C8—H8A	0.9700
N2—C10	1.493 (9)	C8—H8B	0.9700
N2—H2A	0.9100	C9—H9A	0.9700
N3—C13	1.152 (6)	C9—H9B	0.9700
O1—C4	1.300 (6)	C10—C11	1.448 (11)
S1—C13	1.639 (6)	C10—C12	1.458 (12)
Br1—C1	1.905 (5)	C10—H10	0.9800
C1—C2	1.364 (7)	C11—H11A	0.9600
C1—C6	1.394 (8)	C11—H11B	0.9600
C2—C3	1.406 (7)	C11—H11C	0.9600
C2—H2	0.9300	C12—H12A	0.9600
C3—C4	1.428 (7)	C12—H12B	0.9600
C3—C7	1.436 (7)	C12—H12C	0.9600
			
O1—Cu1—N1	92.32 (17)	N1—C7—C3	124.6 (5)
O1—Cu1—N3	87.98 (19)	N1—C7—H7	117.7
N1—Cu1—N3	177.5 (2)	C3—C7—H7	117.7
O1—Cu1—N2	171.59 (18)	N1—C8—C9	108.5 (5)
N1—Cu1—N2	84.45 (18)	N1—C8—H8A	110.0
N3—Cu1—N2	94.91 (19)	C9—C8—H8A	110.0
C7—N1—C8	120.1 (4)	N1—C8—H8B	110.0
C7—N1—Cu1	126.3 (3)	C9—C8—H8B	110.0
C8—N1—Cu1	113.5 (3)	H8A—C8—H8B	108.4
C9—N2—C10	115.9 (5)	N2—C9—C8	108.5 (5)
C9—N2—Cu1	106.1 (3)	N2—C9—H9A	110.0
C10—N2—Cu1	120.5 (4)	C8—C9—H9A	110.0
C9—N2—H2A	104.2	N2—C9—H9B	110.0
C10—N2—H2A	104.2	C8—C9—H9B	110.0
Cu1—N2—H2A	104.2	H9A—C9—H9B	108.4
C13—N3—Cu1	162.4 (5)	C11—C10—C12	116.1 (9)
C4—O1—Cu1	126.3 (3)	C11—C10—N2	113.2 (7)
C2—C1—C6	119.7 (5)	C12—C10—N2	112.3 (6)
C2—C1—Br1	122.8 (4)	C11—C10—H10	104.6
C6—C1—Br1	117.5 (4)	C12—C10—H10	104.6
C1—C2—C3	121.6 (5)	N2—C10—H10	104.6
C1—C2—H2	119.2	C10—C11—H11A	109.5
C3—C2—H2	119.2	C10—C11—H11B	109.5
C2—C3—C4	120.1 (5)	H11A—C11—H11B	109.5
C2—C3—C7	118.1 (5)	C10—C11—H11C	109.5
C4—C3—C7	121.8 (5)	H11A—C11—H11C	109.5
O1—C4—C5	118.8 (5)	H11B—C11—H11C	109.5
O1—C4—C3	125.2 (5)	C10—C12—H12A	109.5
C5—C4—C3	116.0 (5)	C10—C12—H12B	109.5
C6—C5—C4	122.4 (5)	H12A—C12—H12B	109.5
C6—C5—H5	118.8	C10—C12—H12C	109.5
C4—C5—H5	118.8	H12A—C12—H12C	109.5
C5—C6—C1	120.2 (5)	H12B—C12—H12C	109.5
C5—C6—H6	119.9	N3—C13—S1	178.7 (6)
C1—C6—H6	119.9		
